# Identification of a novel *Candida metapsilosis* isolate reveals multiple hybridization events

**DOI:** 10.1093/g3journal/jkab367

**Published:** 2021-10-25

**Authors:** Caoimhe E O’Brien, Bing Zhai, Mihaela Ola, Sean A Bergin, Eoin Ó Cinnéide, Ísla O’Connor, Thierry Rolling, Edwin Miranda, N Esther Babady, Tobias M Hohl, Geraldine Butler

**Affiliations:** School of Biomolecular and Biomedical Science, Conway Institute, University College Dublin, Dublin 4, Ireland; Infectious Disease Service, Department of Medicine, Memorial Sloan Kettering Cancer Center, New York, NY 10065, USA; Immunology Program, Sloan Kettering Institute, Memorial Sloan Kettering Cancer Center, New York, NY 10065, USA; School of Biomolecular and Biomedical Science, Conway Institute, University College Dublin, Dublin 4, Ireland; School of Biomolecular and Biomedical Science, Conway Institute, University College Dublin, Dublin 4, Ireland; School of Medicine, Conway Institute, University College Dublin, Dublin 4, Ireland; School of Biomolecular and Biomedical Science, Conway Institute, University College Dublin, Dublin 4, Ireland; Infectious Disease Service, Department of Medicine, Memorial Sloan Kettering Cancer Center, New York, NY 10065, USA; Immunology Program, Sloan Kettering Institute, Memorial Sloan Kettering Cancer Center, New York, NY 10065, USA; Infectious Disease Service, Department of Medicine, Memorial Sloan Kettering Cancer Center, New York, NY 10065, USA; Immunology Program, Sloan Kettering Institute, Memorial Sloan Kettering Cancer Center, New York, NY 10065, USA; Infectious Disease Service, Department of Medicine, Memorial Sloan Kettering Cancer Center, New York, NY 10065, USA; Immunology Program, Sloan Kettering Institute, Memorial Sloan Kettering Cancer Center, New York, NY 10065, USA; Infectious Disease Service, Department of Medicine, Memorial Sloan Kettering Cancer Center, New York, NY 10065, USA; Immunology Program, Sloan Kettering Institute, Memorial Sloan Kettering Cancer Center, New York, NY 10065, USA; Department of Medicine, Weill Cornell Medical College, New York, NY 10007, USA; School of Biomolecular and Biomedical Science, Conway Institute, University College Dublin, Dublin 4, Ireland

**Keywords:** *Candida*, genomics, hybridization, LOH, mating type-like loci

## Abstract

*Candida metapsilosis* is a member of the *Candida parapsilosis* species complex, a group of opportunistic human pathogens. Of all the members of this complex, *C. metapsilosis* is the least virulent, and accounts for a small proportion of invasive *Candida* infections. Previous studies established that all *C. metapsilosis* isolates are hybrids, originating from a single hybridization event between two lineages, parent A and parent B. Here, we use MinION and Illumina sequencing to characterize a *C. metapsilosis* isolate that originated from a separate hybridization. One of the parents of the new isolate is very closely related to parent A. However, the other parent (parent C) is not the same as parent B. Unlike *C. metapsilosis* AB isolates, the *C. metapsilosis* AC isolate has not undergone introgression at the mating type-like locus. In addition, the A and C haplotypes are not fully collinear. The *C. metapsilosis* AC isolate has undergone loss of heterozygosity with a preference for haplotype A, indicating that this isolate is in the early stages of genome stabilization.

## Introduction

*Candida metapsilosis* is a rare opportunistic pathogen of humans (Gomez-Lopez *et al.* 2008; Lockhart *et al.* 2008a; Silva *et al.* 2009). It is a member of the *Candida parapsilosis* species complex, a small clade of related organisms that includes *C. metapsilosis*, *Candida orthopsilosis* and *C. parapsilosis* sensu stricto, all of which cause infection in humans (Tavanti *et al.* 2005). Within this group, *C. parapsilosis* is the most common cause of candidiasis, whereas *C. metapsilosis* is the least common, with an incidence ranging from 0.6% to 6.9% of cases of invasive candidiasis (Gomez-Lopez *et al.* 2008; Lockhart *et al.* 2008a; Silva *et al.* 2009; Canton *et al.* 2011; Bonfietti *et al.* 2012; Bertini *et al.* 2013). However, isolates from the *C. parapsilosis* species complex are commonly misidentified, which may have led to an underestimation of the frequency of *C. orthopsilosis* and *C. metapsilosis* (Tavanti *et al.* 2005; Lockhart *et al.* 2008b; Bonfietti *et al.* 2012). In recent years, it has been suggested that the incidence of *C. orthopsilosis* and *C. metapsilosis* infection is increasing, although this may be due to increased awareness of the species differentiation (Lockhart *et al.* 2008b). 

Few *C. metapsilosis* isolates secrete virulence-associated factors such as lipases or aspartic proteinases in comparison to *C. parapsilosis* (Nemeth *et al.* 2013). Whereas, *C. parapsilosis* sensu stricto is commonly associated with infections in neonates, *C. metapsilosis* is rarely associated with neonatal infection and appears to affect adults predominantly (Canton *et al.* 2011). There is no evidence of widespread resistance to any antifungal drugs in *C. metapsilosis* and most isolates tested thus far are susceptible to antifungals (Gomez-Lopez *et al.* 2008). There is some suggestion that *C. metapsilosis* may be a human commensal, and it has been isolated from the oral cavity of healthy individuals (Ghannoum *et al.* 2010).

Although all members of the *C. parapsilosis* species complex have diploid genomes, *C. parapsilosis* isolates are highly homozygous with, on average, 0.06 heterozygous SNPs per kb (Butler *et al.* 2009; Pryszcz *et al.* 2013), whereas the majority of isolates of *C. orthopsilosis* and *C. metapsilosis* are extremely heterozygous (Pryszcz *et al.* 2014; Schroder *et al.* 2016). Most *C. orthopsilosis* isolates have heterozygosity levels ranging from 8 to 31 SNPs per kilobase, and originated from multiple hybridization (mating) events between related parents (Pryszcz *et al.* 2014; Schroder *et al.* 2016). Previous analysis of 11 *C. metapsilosis* isolates showed that they were highly heterozygous, ranging from 22 to 26 SNPs per kilobase (Pryszcz *et al.* 2015). The authors proposed that these *C. metapsilosis* isolates arose from hybridization between two parental lineages that differed by approximately 4.5% divergence at the genome level.

Ten of the 11 *C. metapsilosis* isolates previously analyzed by Pryszcz *et al.* (2015) are heterozygous at the mating type-like locus, with both *MTL***a** and *MTLα* idiomorphs present. The *MTLα* locus is intact, and is identical in the arrangement and orientation of its genes to the *MTLα* locus in *C. albicans, C.* *tropicalis*, and *C. orthopsilosis* (Pryszcz *et al.* 2015). However, introgression has occurred at the *MTL***a** locus, where the *PAP***a**, *OBP***a**, and *PIK***a** genes present in most *Candida* species have been overwritten with *MTLα*2, *OBPα*, and a portion of *PIKα*. Because the introgression is present in almost all sequenced *C. metapsilosis* isolates, it is likely that hybridization occurred once, followed by introgression, and all extant isolates descended from this. The 11th *C. metapsilosis* isolate is missing all of *MTL***a**, which Pryszcz *et al.* (2015) proposed resulted from an additional loss of heterozygosity (LOH) event that has overwritten the remainder of the cassette.

Many fungal species that infect humans are hybrids, including *C. orthopsilosis* (Pryszcz *et al.* 2014; Schroder *et al.* 2016), *Candida inconspicua* (Mixao *et al.* 2019) and some *C. tropicalis* strains (O'Brien *et al.* 2021). In some human fungal pathogens, such as *Cryptococcus neoformans* (Li *et al.* 2012) and *Aspergillus* species (Steenwyk *et al.* 2020), and in fungal plant pathogens, hybridization is associated with increased virulence, increased antifungal resistance or an expanded host range (reviewed in Stukenbrock 2016; Mixao and Gabaldon 2018). Hybridization is associated with speciation, such as the emergence of the grass pathogen *Zymoseptoria pseudotritici* (Stukenbrock *et al.* 2012). In *C. orthopsilosis* (Schroder *et al.* 2016) and *C.* *neoformans* (Xu *et al.* 2002; Li *et al.* 2012), there is evidence for multiple and possibly ongoing hybridization events. Here, we describe the discovery of a novel hybrid of *C. metapsilosis* isolated from human feces. This isolate originated from a hybridization event between one parent that is similar to one of the parents of the previously sequenced isolates, and a second parent that is approximately 4.7% different. We therefore propose that multiple hybridization events have occurred in the *C. metapsilosis* lineage.

## Materials and methods

### DNA extraction and Illumina sequencing

The isolates used in this study are shown in Supplementary Table S1. Strains from Memorial Sloan Kettering Cancer Center were cultured on Sabouraud (SAB) agar for 48 h at 37°C, then grown in overnight culture in 2–3 ml of Yeast Extract-Peptone-Dextrose (YPD) broth at 240 rpm. Genomic DNA was extracted and DNA libraries were sequenced on an Illumina HiSeq platform generating 100 bp paired-end reads, as described in Zhai *et al.* (2020). Some isolates were previously described in Zhai *et al.* (2020). Illumina data from *C. metapsilosis* strain ATCC 96143 were downloaded from the NCBI Sequence Read Archive (SRA) under the BioProject ID PRJNA432377 (Oh *et al.* 2019). Illumina reads from Pryszcz *et al.* (2015) were downloaded from the SRA under BioProject ID PRJEB1698. The quality of all Illumina data was checked using FastQC (https://www.bioinformatics.babraham.ac.uk/projects/fastqc/). Reads were trimmed with Skewer version 0.2.2, using parameters “-m pe” (paired-end mode) “-l 50” (minimum read length allowed after trimming is 50 bases) “-q 15” (trim 3’ end until the quality of 15 is reached) “-Q 15” (lowest mean quality allowed before trimming) (Jiang *et al.* 2014). Data were assembled using SPAdes version 3.13.1 with the -careful parameter (Bankevich *et al.* 2012).

### MinION sequencing

*Candida* *metapsilosis* MSK414 was cultured on YPD agar for 48 h at 30°C, then grown overnight in 50 ml YPD broth at 30°C, 200 rpm. DNA was extracted using the QIAGEN Genomic-Tip (100/G) kit as per kit instructions. DNA quality was checked with NanoDrop and quantified using the Qubit fluorometer. Libraries were prepared for MinION sequencing and barcoded with the Rapid Barcoding Kit (SQK-RBK004) from Oxford Nanopore Technologies (ONT). Prepared libraries from three species were pooled and loaded onto an ONT MinION flow cell (FLO-MIN106) for sequencing for 50 h. Basecalling for the MinION data was performed using the ONT Guppy software, version 3.2.4+d9ed22f with the following parameters; “–input_path fast5 –save_path fastq –flowcell FLO-MIN106 –kit SQK-RBK004 –verbose_logs –cpu_threads_per_caller 5 –num_callers 7.” Basecalled data were demultiplexed using qcat version 1.1.0 with the following parameters; “–fastq fastq/all_multiplexed_reads.fastq –barcode_dir demultiplex_qcat –detect-middle –min-read-length 1 –trim –kit RBK004 –epi2me.” After demultiplexing, 2.79 Gb of data were assigned to *C. metapsilosis* MSK414. Read quality was checked with NanoPlot version 1.23.1 (De Coster *et al.* 2018). Potential contaminant reads (*i.e.*, any reads mapping to the other species sequenced in multiplex on the flow cell) were identified by command-line BLASTN from the BLAST+ package version 2.2.31 (Camacho *et al.* 2009) and reads with Q < 7 and length <1 kilobase (kb) were removed with NanoFilt version 2.3.0 (De Coster *et al.* 2018). Postfiltering, 2.76 Gb of data assigned to *C. metapsilosis* MSK414 were available for analysis.

Filtered MinION reads were assembled using Canu version 1.8 using recommended parameters for haplotype separation; -p canu_run2 -d canu_run2 genomeSize=13511817 corOutCoverage=200 “batOptions=-dg 3 -db3 -dr 1 -ca 500 -cp 50” -nanopore-raw all_q7l1k.fastq (Koren *et al.* 2017). Illumina reads were used to polish the assembly with Pilon version 1.23 (Walker *et al.* 2014). Assembly statistics were checked using QUAST version 4.6.1 (Gurevich *et al.* 2013). The final assembly consisted of 45 contigs, with 18 chromosomal-sized contigs, an N50 of 1.78 Mb, and an L50 of 6 (Supplementary Table S2). Zeros were removed from the beginning of contig names for clarity. Circos version 0.69 (Krzywinski *et al.* 2009) and Circoletto (Darzentas 2010) were used to visualize alignments.

Each contig in the *C. metapsilosis* MSK414 assembly was assigned to either the A or C parent based on its percentage identity to the best hit in the assembly of *C. metapsilosis* ATCC 96143 (Oh *et al.* 2019). Global percentage identity was measured using MUMmer dnadiff version 1.3 with default options (Kurtz *et al.* 2004). Pairs of contigs mapping to one contig in the *C. metapsilosis* ATCC 96143 reference assembly were assigned as alternative haplotypes of the same chromosome.

### Variant calling and filtering

Variants were called from the Illumina data. Trimmed reads were aligned to the chimeric reference assembly produced by Pryszcz *et al.* (2015). Reads were aligned using bwa mem version 0.7.12-r1039 with default parameters (Li and Durbin 2010). Duplicated read alignments were removed using PicardTools MarkDuplicates version 1.95. Variants [including single nucleotide polymorphisms (SNPs) and insertions/deletions (indels)] were called using the Genome Analysis Toolkit (GATK) HaplotypeCaller version 3.7 with default parameters (McKenna *et al.* 2010). Variants were filtered by removing clusters of variants (5 or more variants within 20 bases) using the GATK VariantFiltration tool with parameters “–clusterSize 5 –clusterWindowSize 20.” Variants were subsequently filtered for genotype quality (GQ) < 20 and depth of coverage (DP) < 10 using GATK VariantFiltration with parameters “–genotypeFilterExpression ‘GQ < 20’ –genotypeFilterName GQFilter –genotypeFilterExpression ‘DP < 10’ –genotypeFilterName DPFilter.”

For SNP trees, variants were called using the GATK HaplotypeCaller version 3.7 (McKenna *et al.* 2010) with the additional parameter “–emitRefConfidence GVCF” to produce GVCF files. Joint genotyping was performed for GVCF files from 42 *C. metapsilosis* strains (Supplementary Table S1) using the GATK GenotypeGVCFs tool with default parameters. SNPs were extracted from the multisample VCF and filtered as described above. Repeated random haplotype sampling (RRHS) was used to randomly choose an allele at all heterozygous variant sites and generate a FASTA sequence of all SNPs for each sample (Lischer *et al.* 2014). This process was completed 1000 times to capture the full breadth of allelic variation in the isolates. Phylogenetic trees were constructed with RAxML version 8.2.9 with the GTRGAMMA model for each of the 1000 SNP sets (Stamatakis 2014). The tree with the best maximum likelihood score was selected as the reference tree, and the remaining 999 trees were used as pseudo-bootstrap trees to generate a supertree.

### Comparison of MTL loci

*MTLα* sequences for *C. orthopsilosis* were obtained from accession number HQ696682.1 (Sai *et al.* 2011). Bases 6900–9726 of *C. orthopsilosis MTL***a** from accession number HQ696681.1 (Sai *et al.* 2011) were compared to the equivalent regions in the other species. ClustalX (Thompson *et al.* 2002) was used to align the sequences and to calculate the percentage identities. One representative isolate (MSK403) from 25 *C. metapsilosis* isolates from a single patient was included (Zhai *et al.* 2020).

### Loss of heterozygosity

Heterozygous regions were defined as regions containing at least two heterozygous variants within 100 base pairs (bp) of each other (Pryszcz *et al.* 2015). Other regions were designated as homozygous. LOH in *C. metapsilosis* MSK414 was further annotated by aligning Illumina reads to the contigs assigned to the A parent from the Canu assembly with BWA-MEM and calling variants with GATK with parameters as described in “Variant calling and filtering” (McKenna *et al.* 2010). LOH regions were annotated as originating from the C parent if they contained at least one homozygous variant, and as originating from the A parent if there were no homozygous variants in the region. LOH blocks were plotted using the karyoploteR package in R (Gel and Serra 2017). Divergence between the haplotypes of *C. metapsilosis* MSK414 was calculated as the number of heterozygous variant sites in heterozygous regions of the genome divided by the total length of all heterozygous regions of the genome.

### Circos plots

To compare the haplotypes of the diploid *C. metapsilosis* MSK414 assembly, contigs were first assigned to haplotypes A and C using BLASTN. Circoletto (with Circos version 0.69) (Darzentas 2010) was used to align the 9 largest contigs assigned to haplotype A to the 9 largest contigs from haplotype C to generate a Circos plot (Krzywinski *et al.* 2009), with options “–e_value 1e-180 –gep 3 –max_ribbons 10000 –hide_orient_lights –z_by alnlen –untangling_off.” To compare the assembly of *C. metapsilosis* ATCC 96143 to the diploid *C. metapsilosis* MSK414 assembly, the eight largest contigs from the *C. metapsilosis* ATCC 96143 reference were aligned to the 18 largest contigs from *C. metapsilosis* MSK414 (including haplotypes A and C), with Circoletto. The same procedure was used to compare the 10 largest contigs from the *C. metapsilosis* chimeric reference assembly to the 18 largest contigs from *C. metapsilosis* MSK414 (including haplotypes A and C).

## Results

### Population study of *C. metapsilosis*

The genomes of 11 *C. metapsilosis* isolates were first sequenced in 2015 (Pryszcz *et al.* 2015). All were hypothesized to originate from mating between two related, but genetically distinct, individuals. The two parents differed from each other by ∼4.5% at the genome level. A haploid chimeric reference assembly that comprised 57 contigs totaling 13.4 Mb was constructed by combining data from two strains (Pryszcz *et al.* 2015). Subsequently, a collapsed haploid assembly was generated from MinION long-read data from *C. metapsilosis* 96413 (Oh *et al.* 2019). Here, we carried out a population genomics analysis of 42 *C. metapsilosis* isolates, including 11 from Pryszcz *et al.* (2015), 1 from Oh *et al.* (2019), and 30 from Memorial Sloan Kettering Cancer Center (MSK), of which 25 were described previously (Zhai *et al.* 2020; Supplementary Table S1). Most of the MSK strains were collected as part of a study of a cohort of adult patients with culture-proven fungal bloodstream infections following allogeneic hematopoietic stem cell transplant (allo-HCT). Among these, 26 *C. metapsilosis* isolates were isolated from a single patient (Zhai *et al.* 2020). Four were isolated from two other patients with different cancers. DNA was sequenced on an Illumina HiSeq to at least 70X coverage.

**Figure 1:**
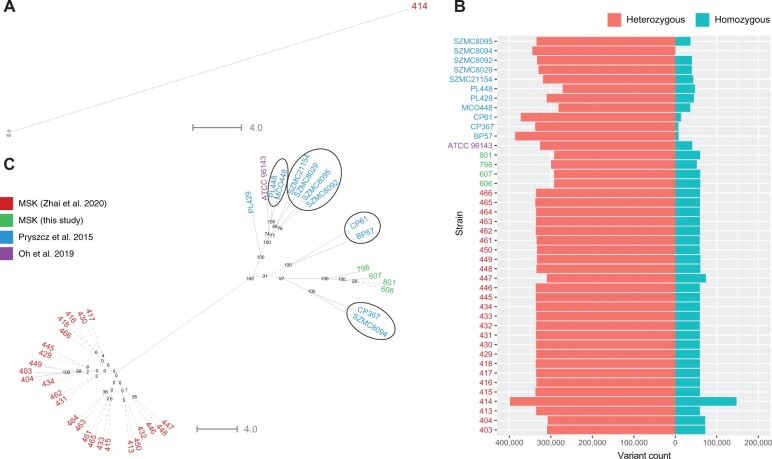
Identification of a divergent *C. metapsilosis* isolate. (A) *Candida metapsilosis* MSK414 is highly divergent. Phylogenetic SNP trees were generated for 42 clinical *C. metapsilosis* isolates from various geographical regions (Supplementary Table S1). SNPs were called using GATK HaplotypeCaller and filtered to remove clusters of variants (5 or more variants within 20 bases) and variants with GQ < 20 or depth of coverage (DP) < 10 using the GATK VariantFiltration tool. RRHS was used to randomly choose an allele at all heterozygous variant sites and generate a FASTA sequence of all SNPs for each sample (Lischer *et al.* 2014). In the case of homozygous SNPs, the alternate allele was chosen by default. This process was repeated 1000 times and 1000 phylogenetic trees were constructed with RAxML using the GTRGAMMA model (Stamatakis 2014). The tree with the best maximum likelihood score was selected as the reference tree, and the remaining 999 trees were used as pseudo-bootstrap trees to generate a supertree. Pseudo-bootstrap values are shown as branch labels. *Candida metapsilosis* MSK414 is labeled in red, while other isolates are not labeled. (B) *Candida metapsilosis* MSK414 has more variants than any other *C. metapsilosis* isolate. Variant count is shown on the bidirectional *x*-axis, with heterozygous variants shown on the left in orange and homozygous variants shown on the right in blue. *Candida metapsilosis* strains are labeled on the *y*-axis. Isolates from MSK are labeled without the “MSK” prefix. Heterozygosity levels range from 271,440 to 398,389 heterozygous variants. *Candida metapsilosis* MSK414 has more heterozygous variants than all other isolates. Some isolates have almost no homozygous variants, *e.g.*, *C. metapsilosis* isolates SZMC8094 (used to construct the reference assembly), CP61, CP367, and BP57. *Candida metapsilosis* MSK414 has more heterozygous variants and more than double the number of homozygous variants of any other *C. metapsilosis* isolate. (C) Other *C. metapsilosis* isolates fall into two main clusters. Phylogenetic trees for all *C. metapsilosis* isolates except MSK414 were drawn as in part A. Isolates from MSK are labeled without the “MSK” prefix. Isolates described by Zhai *et al.* (2020) cluster together and are highly similar. Their relationships cannot be accurately resolved (indicated by dashed lines, bootstrap < 40). Four MSK isolates, *C. metapsilosis* MSK606, *C. metapsilosis* MSK607, *C. metapsilosis* MSK798, and *C. metapsilosis* MSK801, cluster together and are more similar to the clinical isolates described by Pryszcz *et al.* (2015) and Oh *et al.* (2019) than the other isolates from MSK. The inferred phylogenetic relationships of the isolates analyzed by Pryszcz *et al.* (2015) fall into four groups, supporting the original analysis, represented by black circles.

The phylogenetic relationship of the *C. metapsilosis* isolates (Figure  1) was determined by constructing trees using SNPs identified across all 42 isolates relative to the chimeric reference genome constructed by Pryszcz *et al.* (2015). Indels were excluded from this analysis. For heterozygous variant sites, one allele was chosen at random using RRHS (Lischer *et al.* 2014). At homozygous variant sites, the alternative allele to the reference was chosen by default. All variant sites were concatenated and SNP trees were drawn using RAxML (Stamatakis 2014). All isolates have high levels of heterozygosity, ranging from 1 heterozygous variant (*i.e.*, heterozygous SNP or indel) per 34 bp to 1 per 49 bp, with an average of 1 per 41 bp (Figure  1B). *Candida* *metapsilosis* MSK414 is distantly related to all other *C. metapsilosis* isolates (Figure  1A). It is also the most heterozygous isolate analyzed, with 398,389 heterozygous variants (1 per 34 bp) (Figure  1B). *Candida* *metapsilosis* MSK414 also has a high number of homozygous variants compared to the *C. metapsilosis* chimeric reference assembly (Pryszcz *et al.* 2015). On average, the *C. metapsilosis* isolates have 53,799 homozygous variants (standard deviation: 22,478), whereas *C. metapsilosis* MSK414 has 147,375 homozygous variants (Figure  1B).

To facilitate a comparison among the other *C. metapsilosis* isolates, SNP trees were drawn excluding *C. metapsilosis* MSK414 (Figure  1C). Twenty-five of the twenty-six *C. metapsilosis* MSK strains (designated by four as the first digit) isolated from a single patient cluster together, as described previously (Zhai *et al.* 2020). The genomes of these isolates are highly similar (Figure  1C) and could not be differentiated by phylogenetic analysis, although there are some differences in homozygosity levels (Supplementary Figure S1). Four additional *C. metapsilosis* isolates from MSK (labeled in green on Figure  1C) cluster separately from the other MSK strains. Isolates described by Pryszcz *et al.* (2015) fall into approximately four clades (encircled in Figure  1C), as previously described. *Candida* *metapsilosis* PL429 does not belong to any clade. *Candida* *metapsilosis* ATCC 96143, a clinical isolate from Livermore, USA, clusters with one of the groups previously identified by Pryszcz *et al.* (2015), together with *C. metapsilosis* MCO448 and *C. metapsilosis* PL448, which are both clinical isolates from Washington, USA.

### Identification of a novel *C. metapsilosis* hybrid

Figure  1A shows that *C. metapsilosis* MSK414 is very different to the other *C. metapsilosis* isolates. We therefore attempted to assemble its genome to facilitate comparison. Previous studies have shown that there are many limitations associated with assembly of short-read data from heterozygous diploids (Chan *et al.* 2012; Zheng *et al.* 2013; Pryszcz and Gabaldon 2016). During assembly of most diploid genomes, the two haplotypes collapse into a single contig, yielding a haploid assembly. However, for highly heterozygous genomes, this is not possible, and the resulting assemblies are highly fragmented (Pevzner *et al.* 2001; Li *et al.* 2010; Gnerre *et al.* 2011). Pryszcz and Gabaldón (2016) developed a protocol (Redundans) that produces a haploid reference assembly by collapsing sequence information from both haplotypes. At heterozygous sites, one allele is randomly chosen to generate one representative contig per diploid chromosome. They assembled a chimeric *C. metapsilosis* haploid genome, using data from two isolates, that has 57 contigs (Pryszcz *et al.* 2015). However, haplotype information has been lost from this assembly.

**Figure 2:**
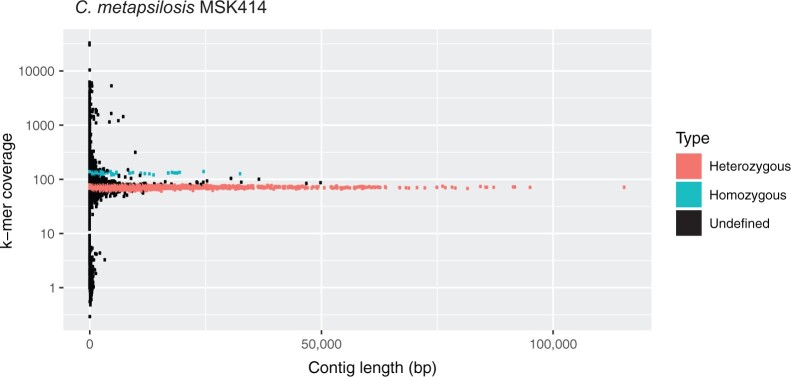
Illumina assembly of *C. metapsilosis* MSK414 reveals two peaks of coverage. Scaffolds from the SPAdes assembly of the Illumina data from *C. metapsilosis* MSK414 are shown as dots. Scaffold length is shown on the *x*-axis and scaffold k-mer coverage is shown on the *y*-axis on a log scale. The majority of the scaffolds have approximately 70X coverage (red). These scaffolds represent heterozygous regions where both haplotypes have been assembled separately. A second peak of coverage is visible at approximately 130X (cyan). These scaffolds represent homozygous regions that have been collapsed. This structure suggests that *C. metapsilosis* MSK414 has a hybrid genome (*i.e.*, the two haplotypes are distinctly different).

We used SPAdes (Bankevich *et al.* 2012), which keeps haplotypes separate, to assemble the genomes of 42 *C. metapsilosis* isolates (Supplementary Table S1). Scaffolds fell into two groups, where the depth of coverage of one group was approximately half of the coverage of the second group. Scaffolds with half coverage represent heterozygous regions where both haplotypes have been assembled separately. Scaffolds with high depth of coverage represent homozygous regions that have been collapsed into a single scaffold. This is shown for *C. metapsilosis* MSK414 (Figure  2) using a coverage-*vs*-length plot (Douglass *et al.* 2019). This assembly pattern suggests that like all other *C. metapsilosis* isolates, *C. metapsilosis* MSK414 is a hybrid.

To improve the assembly of *C. metapsilosis* MSK414, we used Oxford Nanopore MinION long-read sequencing. The reads were assembled using Canu (Koren *et al.* 2017), and errors were corrected by incorporating the Illumina data using Pilon (Walker *et al.* 2014). This generated an assembly of 45 contigs, with 18 larger than 450 kb, totaling 27 Mb (Supplementary Table S2). The contigs smaller than 450 kb were derived from the mitochondrial genome, or from within the chromosomal-sized contigs. A telomeric repeat (ACTTTGGACATCCTAACCTCAAT) was identified at both ends of 14 contigs, and at one end of three of the largest contigs in the assembly. Centromeres (Ola *et al.* 2020) were identified in 16 contigs, which is consistent with hybridization between two parents with eight chromosomes each (Supplementary Table S3).

**Figure 3:**
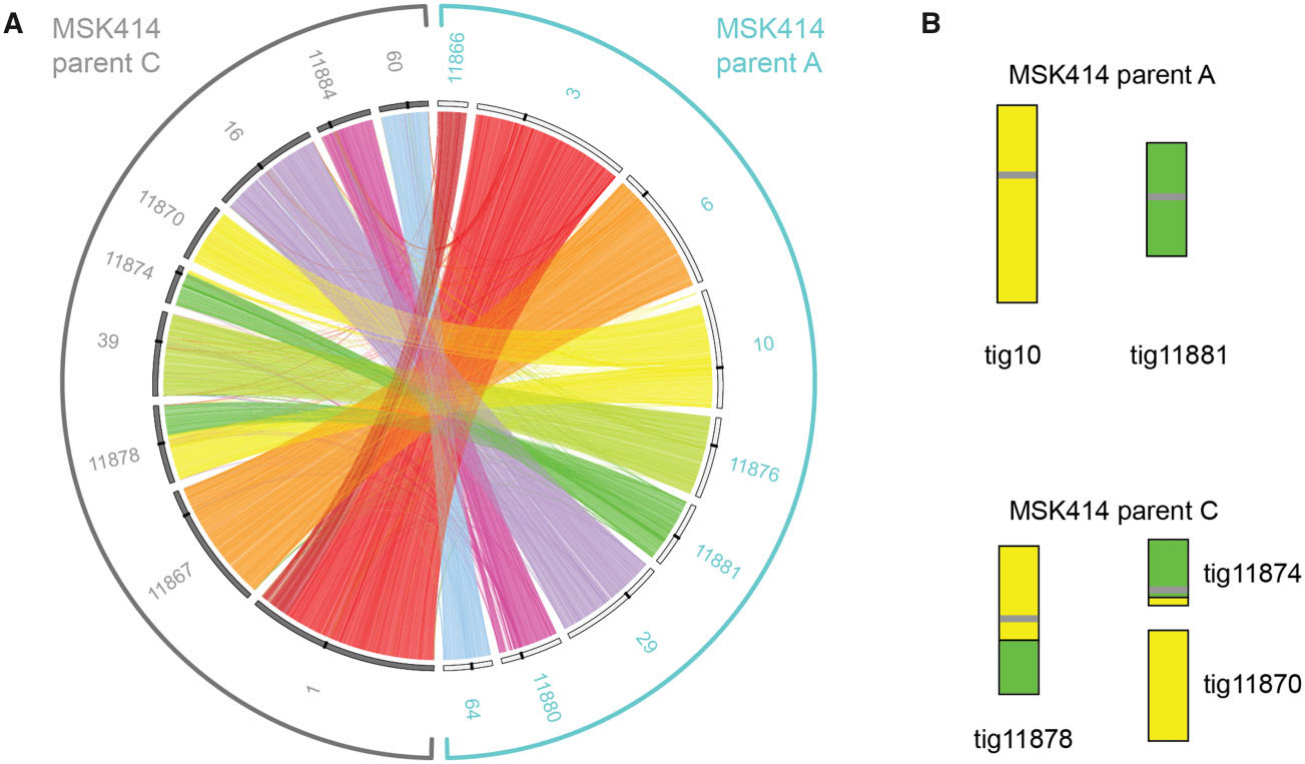
Haplotypes A and C in *C. metapsilosis* MSK414 differ by one reciprocal translocation. (A) Similarity between the haplotypes of *C. metapsilosis* MSK414 was visualized using Circos (Krzywinski *et al.* 2009) and Circoletto (Darzentas 2010). The 18 largest contigs in the assembly are shown, with the haplotype from the putative A parent on the right (outlined in turquoise) and from the putative C parent on the left (outlined in gray). For clarity, contigs are labeled without the “tig” prefix. Sequences with similarity were identified by BLASTN and alignments with the minimum *E*-value (1e-180) were plotted as links between the two haplotypes. The 9 largest contigs from parent A (shared with other *C. metapsilosis* isolates) are shown on the right-hand side with white bars on the inner layer. The nine largest contigs from parent C are shown on the left with gray bars on the inner layer. Centromeres are shown as black bars on the inner layer. A translocation is evident between tig10 and tig11881 in the A haplotype and the equivalent contigs in the C haplotype. (B) Translocation between tig10 and tig11881 from haplotype A in haplotype C of *C. metapsilosis* MSK414. Contigs in the *C. metapsilosis* parent A and C haplotypes are shown as colored bars. Centromeres are shown as gray horizontal bars on the contigs.

To assign the contigs to haplotypes, we compared them to a haploid assembly of *C. metapsilosis* ATCC 96143 (Oh *et al.* 2019). This assembly is more complete (eight scaffolds) than the original chimeric reference assembly generated by Pryszcz *et al.* (2015), but still represents a collapsed haploid. In most cases, there is a 1:2 relationship between the haploid assembly and the *C. metapsilosis* MSK414 contigs (Supplementary Table S3, Figure  3). For each of these, one *C. metapsilosis* MSK414 contig is more similar to the reference (94–96% identity) and one is less similar (92–93%). These likely represent the haplotypes of the original parents of MSK414. Contigs 3.1 and 5.1 of *C. metapsilosis* ATCC 96143 match two contigs in one haplotype of *C. metapsilosis* MSK414 because of the reciprocal translocation (Figure  3B). We assigned the set of contigs that are more similar to *C. metapsilosis* ATCC 96143 as haplotype A, and the set of contigs that are less similar to C. *metapsilosis* ATCC 96143 as haplotype C (Supplementary Table S3).

By comparing the contigs to each other, we found that here is a direct relationship between 13 of the 18 largest contigs (Figure  3). Based on similarities (Figure  3A and Supplementary Table S3) we hypothesize that tig11866 and tig3 should be joined, and they represent the haplotype A equivalent of tig1 from haplotype C (Figure  3). These 13 contigs, therefore, represent both haplotypes for six of the eight pairs of *C. metapsilosis* chromosomes. However, the remaining two chromosome pairs are not collinear. tig10 from one haplotype (haplotype A) matches parts of both tig11870 and tig11878 from the second haplotype (haplotype C) (Figure  3A). Similarly, tig11881 from one haplotype (haplotype A) matches part of tig11878 and tig11874 from the second haplotype (haplotype C). Based on similarities, we hypothesize that tig11870 and tig11874 (haplotype C) should be joined (Figure  3B). This is consistent with a single translocation event in haplotype C (Figure  3B). The translocated chromosomes contain the mating type-like loci (MTL).

### Analysis of the mating type-like locus

**Figure 4:**
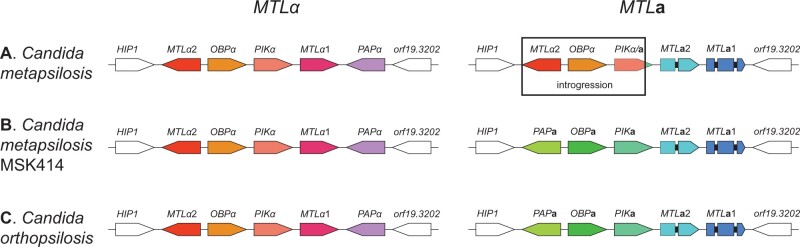
*Candida metapsilosis* MSK414 has a distinct arrangement at *MTL***a**. The structure of the *MTL***a** and MTLα idiomorphs in the majority of *C. metapsilosis* isolates (A), *C. metapsilosis* MSK414 (B), and C. *orthopsilosis* (C) are shown, with their relative orientations. Introns in the *MTL***a1** and *MTL***a2** genes are shown as black bars. In most *C. metapsilosis* isolates, the *MTLα* locus is intact, and is identical in its order and orientation to that of *C. orthopsilosis*. The *MTL***a** locus has been partially overwritten by a portion of the *MTLα* locus containing the genes *MTLα2*, *OBPα*, and a part of *PIKα.* The *PIK* gene is chimeric, comprising part of *PIKα* and part of *PIK***a**. Both MTL idiomorphs in *C. metapsilosis* isolate MSK414 (B) have the same order and orientation as in *C. orthopsilosis* (C). In this isolate, the *MTLα* and *MTL***a** idiomorphs are intact with no introgression.

Pryszcz *et al.* (2015) showed that *MTLα* is intact in 11 *C. metapsilosis* isolates, and is identical in the order and orientation of its genes to the *MTLα* locus in *C. albicans*, *C.* *tropicalis*, and *C. orthopsilosis*. The *MTL***a** locus, however, has been partially overwritten with information from the *MTLα* locus (Figure  4). In *MTL***a**, the *PAP***a** and *OBP***a** genes have been replaced by the *MTLα*2 and *OBPα* genes. A portion of *PIK***a** has been overwritten with a portion of *PIKα*, producing a chimeric *PIK* gene at the *MTL***a**. We found the same *MTL* arrangement in 30 additional *C. metapsilosis* isolates (*C. metapsilosis* ATCC 96143 and 29 *C. metapsilosis* isolates from MSK). However, *C. metapsilosis* MSK414 has a different organization; both *MTL***a** and *MTLα* are intact (Figure  4).

The *MTLα* locus from *C. metapsilosis* MSK414 is ∼99.8% identical to the *MTLα* locus from *C. metapsilosis* ATCC 96143, and to all other sequenced *MTLα* loci (Table  1). In addition, the copy of *orf19.3202* that is adjacent to *MTLα* is 97% identical to the reference genome. We therefore hypothesize that *MTLα* was contributed by the same parent, or a very similar parent, in all previously described *C. metapsilosis* isolates and in *C. metapsilosis* MSK414 (parent A). For most *C. metapsilosis* isolates, a second parent (parent B) donated the *MTL***a** locus, which has subsequently been overwritten. The *MTL***a** loci in most *C. metapsilosis* isolates are very similar (>99.9% identical, Table  1). However, even the region of *MTL***a** that is conserved in *C. metapsilosis* MSK414 shares only ∼91.4% similarity with other *C. metapsilosis* isolates, supporting our hypothesis that in this isolate, *MTL***a** was donated by a third parent, parent C (Table  1). The majority of *C. metapsilosis* isolates are AB hybrids, whereas *C. metapsilosis* MSK414 is an AC hybrid.

**Table 1:** Comparison of sequence identity at MTL loci

	*C. parapsilosis*	*C. orthopsilosis*	***C. metapsilosis*** *^a^*
*C. metapsilosis* MSK414 *MTL***a***^b^*	49.92%	56.62%	91.4% (>99.9%)
*C. metapsilosis* MSK414 *MTLα*	–	72.95%	99.83% (>99%)

a *Candida metapsilosis* isolates other than MSK414. The numbers in parentheses show the similarity within these isolates.

b The short region of *MTL***a** that is not overwritten in most *C. metapsilosis* isolates was used in comparisons.

### Loss of heterozygosity

**Figure 5:**
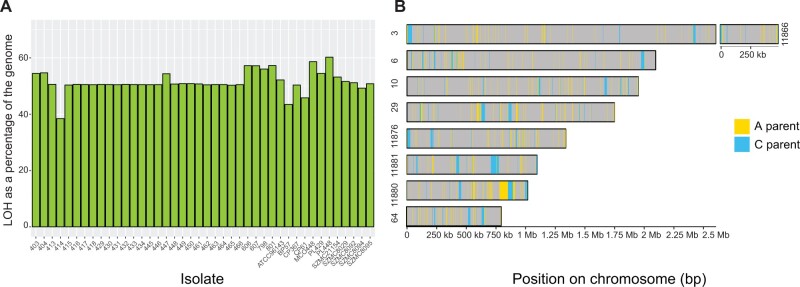
The genome of *C. metapsilosis* MSK414 has undergone less LOH than other *C. metapsilosis* isolates. (A) Bar plot showing the percentage of the genome that has undergone LOH (*y*-axis) in *C. metapsilosis* isolates (*x*-axis). For most isolates, more than 50% of the genome has undergone LOH, equating to approximately 6.7 Mb. Only 38% of the *C. metapsilosis* MSK414 genome has undergone LOH. Two other isolates, *C. metapsilosis* BP57 and *C. metapsilosis* CP61, which form a cluster on the SNP phylogeny, have also undergone less LOH than the other isolates (approximately 43% and 45%, respectively). (B) Regions of LOH in *C. metapsilosis* MSK414 are dispersed throughout the genome. The nine largest contigs assigned to the *C. metapsilosis* MSK414 A haplotype are shown. For the sake of clarity, only LOH regions of at least 1 kb are illustrated here. Heterozygous regions (defined as any region with at least two heterozygous variants within 100 bp of each other), undefined regions and LOH regions less than 1 kb are colored in gray. LOH blocks were defined as any region of at least 100 bp with fewer than two heterozygous variants. LOH regions were assigned to the A parent haplotype (colored in yellow) if there were any homozygous variants present, and to the C parent haplotype (colored in blue) if there were no homozygous variants.

Previous studies observed that *C. metapsilosis* isolates have undergone large-scale LOH events (Pryszcz *et al.* 2015). In addition, we found that *C. metapsilosis* isolates MSK403, MSK404, and MSK447 have undergone LOH across most of scaffold 4 (Supplementary Figure S1). All isolates except for *C. metapsilosis* MSK414 have undergone significant LOH across part of two scaffolds (Supplementary Figure S1), supporting the hypothesis that they all descended from the same ancestor. Each *C. metapsilosis* genome has undergone LOH over approximately half its length (Figure  5A).

The novel hybrid MSK414 stands out as having undergone relatively little LOH (38% of its length; 5.2 Mb) (Figure  5A). Regions of LOH were assigned to either the C parent (at least one homozygous variant in 100 bp) or to the A parent (no homozygous variants). LOH regions assigned to parent C totaled ∼5.4% of the total genome length, while LOH assigned to the A parent totaled ∼26% of the total genome length. There are 350 blocks of LOH with an average length of 251 bp assigned to the C haplotype, and 13,892 blocks with an average length of 2080 bp assigned to the A haplotype. The LOH tracts are randomly dispersed throughout the contigs (Figure  5B). Haplotypes A and C of *C. metapsilosis* MSK414 diverge by approximately 4.71%, based on the number of heterozygous sites in heterozygous regions (*i.e.*, sites that differ between the haplotypes of *C. metapsilosis* MSK414) relative to the total length of all heterozygous regions.

## Discussion

Previously sequenced *C. metapsilosis* isolates have an intact *MTLα* locus, with introgression at *MTL***a** (Pryszcz *et al.* 2015). We found this arrangement in 30 additional isolates from the USA (MSK, and *C. metapsilosis* ATCC 96143). The relative lack of divergence among these isolates (Figure  1B), and the observation that most share LOH tracts, suggest that they are all derived from the same hybrid ancestor. It is likely that a single ancient hybridization event between A and B parents that differ by 4.5% was followed by introgression at *MTL***a**, and that most *C. metapsilosis* isolates descended from this single event.

Despite a lack of evidence at the time, Pryszcz *et al.* (2015) suggested that additional hybrid lineages of *C. metapsilosis* may be found. Indeed, analysis of other fungal species, including *C.* *neoformans* and *C. orthopsilosis*, showed that hybridization in those species has occurred on multiple separate occasions (Xu *et al.* 2002; Li *et al.* 2012; Schroder *et al.* 2016). However, until now, no different hybrids of *C. metapsilosis* have been identified. Our results show that *C. metapsilosis* MSK414 most likely shares one parent (A) with other *C. metapsilosis* isolates, but its second parent (C) is distinctly different. Parent C has donated an intact *MTL***a** idiomorph. The A and C haplotypes differ by approximately 4.7%, similar to the divergence between the A and B haplotypes in the other *C. metapsilosis* isolates (4.5%; Pryszcz *et al.* 2015). This is also similar to the divergence between haplotypes in hybrids of *C. orthopsilosis* (Schroder *et al.* 2016) and *C. tropicalis* (O'Brien *et al.* 2021).

Identification and separation of parental haplotypes in hybrid species is difficult unless at least one of the parents is known (*e.g.*, *C. orthopsilosis*, Riccombeni *et al.* 2012), or if pure lineages of the contributing parents are available. Pure lineages of the A, B, and C haplotypes of *C. metapsilosis* have not yet been identified, but we were able to separate the A and C haplotypes using long-read sequencing (ONT).

Pryszcz *et al.* (2015) suggested that only hybrid lineages of *Candida* species are pathogenic, and that homozygous isolates may only be found in nonclinical samples. This proposal is supported by the observation that most clinical isolates of *C. orthopsilosis* are hybrids (Schroder *et al.* 2016), and that *C. albicans* is an ancient hybrid (Mixao and Gabaldon 2020). However, rare hybrids of *C. tropicalis* are enriched in environmental, and not clinical sites (O'Brien *et al.* 2021). Although *C. metapsilosis* has been isolated from several different body sites, including blood, feces, mucosa, nails, skin, and urine (Hensgens *et al.* 2009), its natural environment is not known. Because all clinical isolates of *C. metapsilosis* are hybrids, it is possible that hybridization of isolates in the environment may have enabled *C. metapsilosis* to colonies a new niche, namely the human host. However, the effect of hybridization on pathogenicity cannot be fully characterized until environmental isolates are identified. In addition, “AC” hybrids are rare (1 of 42 isolates),

Our results, together with studies in other species such as *C. albicans* (Mixao and Gabaldon 2020), *C. orthopsilosis* (Pryszcz *et al.* 2014; Schroder *et al.* 2016), *C. tropicalis* (O'Brien *et al.* 2021), and *Millerozyma sorbitophila* (Louis *et al.* 2012), suggest that hybridization occurs frequently in members of the CUG-Ser1 clade. Hybridization may represent a mode of adaptation to the host, or possibly to other as yet undetermined conditions.

## Data availability

All strains are available by request. The raw Illumina data for *C. metapsilosis* MSK606, MSK607, MSK798, MSK801, and MSK414 are available at BioProject PRJNA748054. The raw MinION data for *C. metapsilosis* MSK414 are available at accession number SRR15054248, and the genome assembly is available at BioProject PRJNA730502 (accession number JAHFZM000000000).

Supplementary material is available at *G3* online.

## Supplementary Material

jkab367_Supplementary_Figure1

jkab367_Supplementary_Figure2

jkab367_Supplementary_Table1

jkab367_Supplementary_Table2

jkab367_Supplementary_Table3

jkab367_Supplementary_Figures-Tables-Captions
