# Supplementary material for: Identification of a novel Candida metapsilosis isolate reveals multiple hybridization events
Source: G3 (Bethesda). 2021 Oct 25;12(1):jkab367. doi: 10.1093/g3journal/jkab367 (PMC8727981; doi:10.1093/g3journal/jkab367)
Supplement: jkab367_Supplementary_Figure1 [file jkab367_supplementary_figure1.pdf]

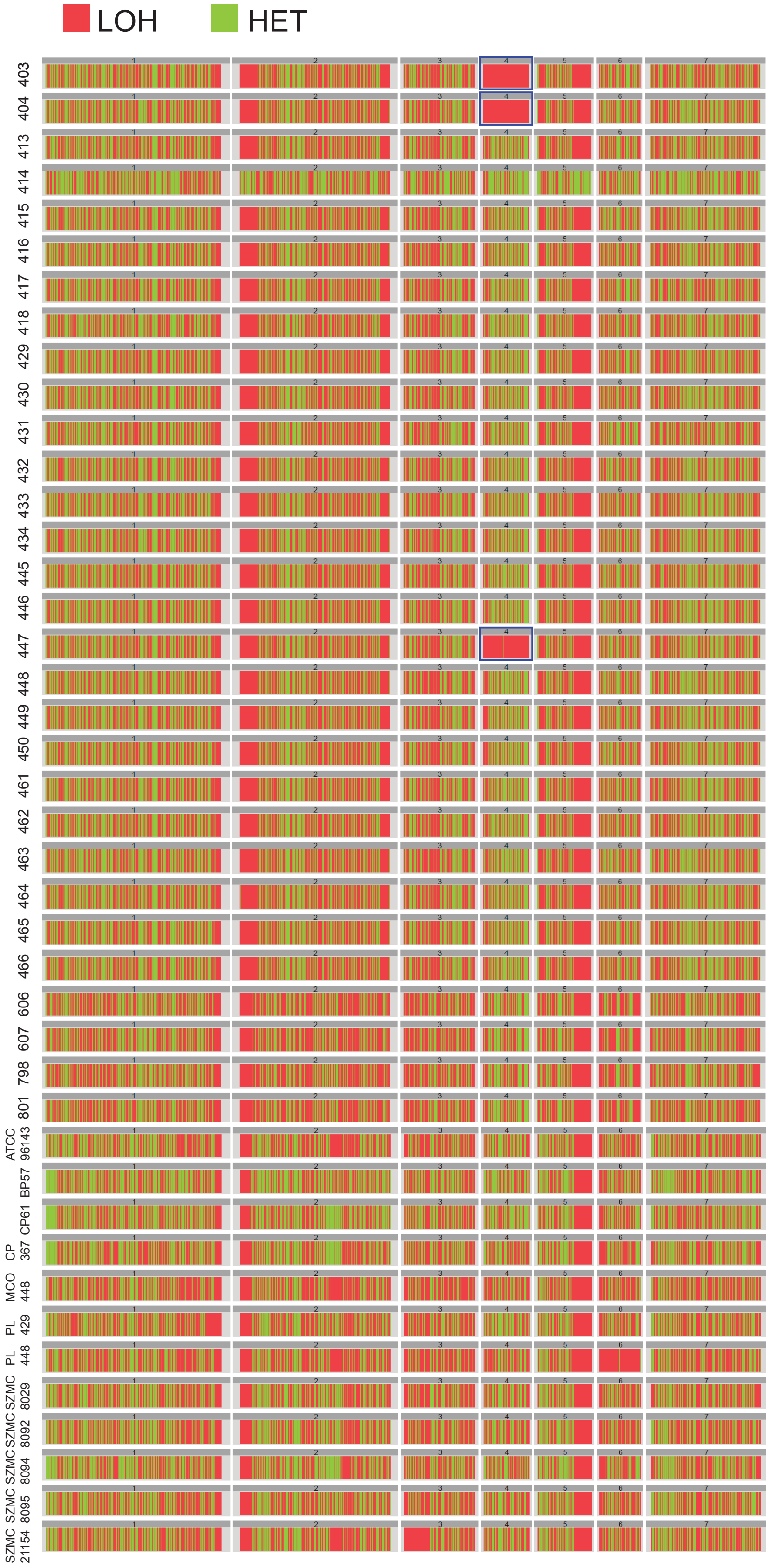

Supplementary Figure 1. Distribution of heterozygous and LOH regions in the genomes of *C. metapsilosis* isolates.

The seven largest scaffolds in the chimeric reference genome are displayed horizontally from left to right and labelled from 1 to 7. Regions of LOH are shown in red and heterozygous (HET) regions are shown in green. Isolates are labelled on the left-hand side. MSK isolates are shown without the "MSK" prefix. The genomes of most isolates consist of a mixture of heterozygous and LOH regions. Isolates 403, 404, and 447 have undergone significant LOH across most of scaffold 4 (highlighted with blue boxes). Isolate PL448 has undergone significant LOH on scaffold 6. Large areas of LOH are visible on scaffolds 2 and 5 in all isolates, except for the new hybrid *C. metapsilosis* MSK414.
