# Supplementary material for: Identification of a novel Candida metapsilosis isolate reveals multiple hybridization events
Source: G3 (Bethesda). 2021 Oct 25;12(1):jkab367. doi: 10.1093/g3journal/jkab367 (PMC8727981; doi:10.1093/g3journal/jkab367)
Supplement: jkab367_Supplementary_Figure2 [file jkab367_supplementary_figure2.pdf]

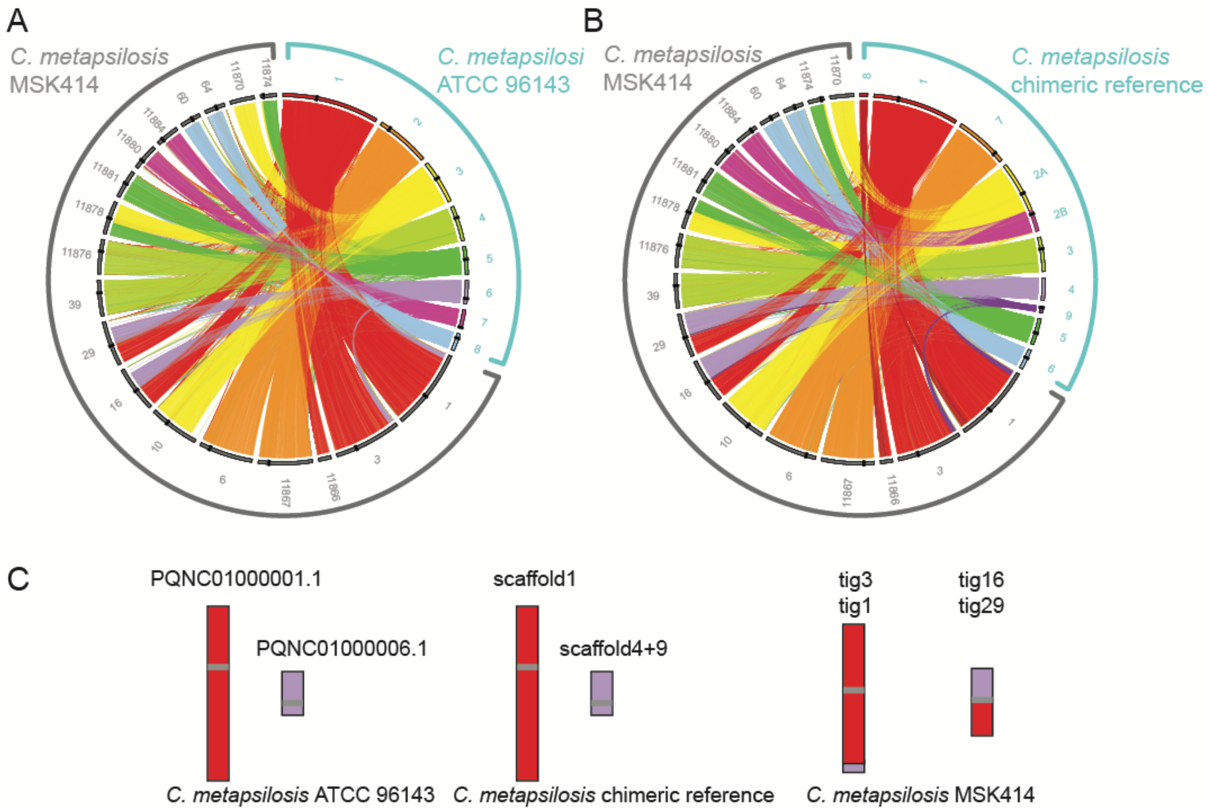

Supplementary Figure 2. Rearrangements between the diploid assembly of C. metapsilosis MSK414 and two haploid C. metapsilosis assemblies.
Sequence similarity between the genome assembly of *C. metapsilosis* MSK414 and **(A)** the assembly of *C. metapsilosis* ATCC 96143, and **(B)** the chimeric reference assembly of *C. metapsilosis*, was visualized using Circos (Krzywinski et al. 2009) and Circoletto (Darzentas 2010). Sequences with similarity were identified by BLASTN and alignments with the minimum E- value (1e-180) were plotted as links between the two assemblies. The 18 largest contigs from the diploid *C. metapsilosis* MSK414 assembly are represented by grey bars and labelled with the “tig” prefix removed for clarity in panels A and B. Centromeres are marked as black bars on the inner layer.

A. Rearrangements between the assembly of *C. metapsilosis* MSK414 and the assembly of *C. metapsilosis* ATCC 96143. The eight largest contigs from the haploid *C. metapsilosis* ATCC 96143 assembly are shown on the upper right-hand side of the Circos plot in panel A with colored bars and outlined in turquoise. A mismatch between PQNC01000001.1 and PQNC01000006.1 is observed in both haplotypes of *C. metapsilosis* MSK414 (tig00000001 and tig00000003, and tig0000016 and tig00000029). A translocation in one haplotype of C. metapsilosis MSK414 is visible between PQNC01000003.1 and PQNC01000005.1 (tig00011878 and tig00011870/tig00011874).

B. Rearrangements between the assembly of *C. metapsilosis* MSK414 and the *C. metapsilosis* chimeric reference assembly. The nine largest contigs from the haploid *C. metapsilosis* ATCC 96143 assembly are shown on the upper right-hand side of the Circos plot in panel B with colored bars and labelled in turquoise. Scaffold

2 contains two centromeres and appears to be the product of an assembly error (supported by the haploid assembly of *C. metapsilosis* ATCC 96143 and tig00000010 of the *C. metapsilosis* MSK414 assembly). We refer these as scaffold 2A and scaffold 2B. A mismatch between scaffold 1 and scaffold 4/9 (which we hypothesize should be joined) is visible in both haplotypes of *C. metapsilosis* MSK414 (tig00000001 and tig00000003, tig00000016 and tig00000029). Scaffolds 4 and 9 most likely represent one chromosome but the assembly has broken at the centromere. This rearrangement mirrors the one between scaffolds 1 and 6 of the *C. metapsilosis* ATCC 96143 assembly.

C. Possible error in the assembly of *C. metapsilosis* ATCC 96143 and the chimeric *C. metapsilosis* reference assembly. Contigs in the *C. metapsilosis* ATCC 96143 assembly, the chimeric *C. metapsilosis* reference assembly and the C. metapsilosis MSK414 Canu assembly are shown as colored bars. Centromeres are shown as grey horizontal bars on the contigs. The rearrangement observed between PQNC010000001.1 and PQNC010000006.1 is equivalent to the rearrangement between scaffold 1 and scaffold 4/9 in the *C. metapsilosis* chimeric reference. This may result from an assembly error in the two haploid assemblies. It may also represent a real difference between the genome of *C. metapsilosis* 414 and the other isolates, though it is unlikely that translocations have occurred in both haplotypes of this isolate.
