# Supplementary material for: Identification of a novel Candida metapsilosis isolate reveals multiple hybridization events
Source: G3 (Bethesda). 2021 Oct 25;12(1):jkab367. doi: 10.1093/g3journal/jkab367 (PMC8727981; doi:10.1093/g3journal/jkab367)
Supplement: jkab367_Supplementary_Table1 [file jkab367_supplementary_table1.docx]

**Table S1. *C. metapsilosis* strains used in this study**

| **Strain ID** | **Origin** | **Site of Isolation** | **Read length** | **Cov** | **Total variants** |
| --- | --- | --- | --- | --- | --- |
| ATCC 96143^1^ | Livermore, USA | Unknown | 150 | 42 | 366,857 |
| BP57**^2^** | Pécs, Hungary | Throat | 96 | 151.31 | 394,081 |
| CP376**^2^** | Pisa, Italy | Feces | 96 | 163.96 | 345,030 |
| CP61**^2^** | Pisa, Italy | Nail | 96 | 151.42 | 386,151 |
| MCO448**^2^** | Washington, USA | Hand | 46 | 175.76 | 317,333 |
| PL429**^2^** | Livermore, USA | Unknown | 76 | 284.81 | 354,922 |
| PL448**^2^** | Washington, USA | Hand | 46 | 186.37 | 318,964 |
| SZMC211154**^2^** | Cataluña, Spain | Blood | 100 | 492.44 | 361,914 |
| SZMC8029**^2^** | Debrecen, Hungary | Blood | 100 | 425.57 | 369,230 |
| SZMC8092**^2^** | Pisa, Italy | Lung | 100 | 470.78 | 373,057 |
| SZMC8094**^2^** | Pisa, Italy | Feces | 100 | 402.91 | 344,840 |
| SZMC8095**^2^** | Pisa, Italy | Nail | 100 | 480.67 | 370,999 |
| MSK403^3^ | New York, USA | Blood | 101 | 205.47 | 381,084 |
| MSK404^3^ | New York, USA | Blood | 101 | 248.1 | 379,288 |
| MSK413^3^ | New York, USA | Feces | 101 | 262.04 | 394,729 |
| MSK414^4^ | New York, USA | Feces | 101 | 258.03 | 545,764 |
| MSK415^3^ | New York, USA | Feces | 101 | 226.01 | 396,420 |
| MSK416^3^ | New York, USA | Feces | 101 | 244.2 | 394,311 |
| MSK417^3^ | New York, USA | Feces | 101 | 285.59 | 394,741 |
| MSK418^3^ | New York, USA | Feces | 101 | 267.76 | 395,401 |
| MSK429^3^ | New York, USA | Feces | 101 | 314.98 | 394,129 |
| MSK430^3^ | New York, USA | Feces | 101 | 293.11 | 394,695 |
| MSK431^3^ | New York, USA | Feces | 101 | 213.65 | 395,459 |
| MSK432^3^ | New York, USA | Feces | 101 | 226.58 | 394,260 |
| MSK433^3^ | New York, USA | Feces | 101 | 257.45 | 394,628 |
| MSK434^3^ | New York, USA | Feces | 101 | 277.28 | 395,131 |
| MSK445^3^ | New York, USA | Feces | 101 | 297.49 | 395,536 |
| MSK446^3^ | New York, USA | Feces | 101 | 314.58 | 395,372 |
| MSK447^3^ | New York, USA | Feces | 101 | 275.69 | 383,033 |
| MSK448^3^ | New York, USA | Feces | 101 | 256.41 | 395,011 |
| MSK449^3^ | New York, USA | Feces | 101 | 284 | 393,777 |
| MSK450^3^ | New York, USA | Feces | 101 | 234.69 | 392,162 |
| MSK461^3^ | New York, USA | Feces | 101 | 257.75 | 393,295 |
| MSK462^3^ | New York, USA | Feces | 101 | 284.83 | 395,788 |
| MSK463^3^ | New York, USA | Feces | 101 | 302.81 | 394,906 |
| MSK464^3^ | New York, USA | Feces | 101 | 287.72 | 394,569 |
| MSK465^3^ | New York, USA | Feces | 101 | 252.57 | 397,063 |
| MSK466^3^ | New York, USA | Feces | 101 | 304.11 | 395,274 |
| MSK606^4^ | New York, USA | Blood | 101 | 344.82 | 352,155 |
| MSK607^4^ | New York, USA | Colon | 101 | 358.94 | 352,121 |
| MSK798^4^ | New York, USA | Blood | 101 | 394.31 | 351,303 |
| MSK801^4^ | New York, USA | Blood | 101 | 413.37 | 351,401 |

^1^ [(Oh *et al.* 2019)](https://paperpile.com/c/FunnkE/JKJW)

^2^ [(Pryszcz *et al.* 2015)](https://paperpile.com/c/FunnkE/dD10)

^3^ [(Zhai *et al.* 2020)](https://paperpile.com/c/FunnkE/tRBe). MSK403-466 were isolated from a single patient.

^4^ This study. MSK606, 607, 798 and 801 were isolated from two patients.
