# Supplementary material for: Identification of a novel Candida metapsilosis isolate reveals multiple hybridization events
Source: G3 (Bethesda). 2021 Oct 25;12(1):jkab367. doi: 10.1093/g3journal/jkab367 (PMC8727981; doi:10.1093/g3journal/jkab367)
Supplement: jkab367_Supplementary_Table2 [file jkab367_supplementary_table2.docx]

**Table S2. Comparison of Illumina and minION assemblies of *C. metapsilosis* MSK414.**

|  | **Illumina (SPAdes)** | **minION (Canu)** |
| --- | --- | --- |
| **Total number of contigs** | 13,527 | 45 |
| **Total length** | 26,170,059 | 27,138,054 |
| **Largest contig** | 115,451 | 3,141,946 |
| **N50** | 22,429 | 1,780,562 |
| **L50** | 337 | 6 |
