# Supplementary material for: Identification of a novel Candida metapsilosis isolate reveals multiple hybridization events
Source: G3 (Bethesda). 2021 Oct 25;12(1):jkab367. doi: 10.1093/g3journal/jkab367 (PMC8727981; doi:10.1093/g3journal/jkab367)
Supplement: jkab367_Supplementary_Table3 [file jkab367_supplementary_table3.docx]

**Table S3. Assignment of contigs in *C. metapsilosis* MSK414 to haplotypes.**

| **ATCC 96143 contig** | **MSK414 parent A** | | **MSK414 parent C** | |
| --- | --- | --- | --- | --- |
| 1.1^1^  3,752,469 | tig3^ct^ | | tig1^tct^ | |
|  | Contig length | 2,603,961 | Contig length | 3,149,771 |
|  | Total length of all alignments | 2,367,064 | Total length of all alignments | 2,798,055 |
|  | Average identity | 94.81 | Average identity | 92.43 |
|  | **Identical bases** | **2,244,213** | **Identical bases** | **2,586,242** |
|  | tig11866^t^ | |  |  |
|  | Contig length | 488,734 |  |  |
|  | Total length of all alignments | 479,695 |  |  |
|  | Average identity | 95.75 |  |  |
|  | **Identical bases** | **459,308** |  |  |
|  | tig29^tct^ | | tig16^tct^ | |
|  | Contig length | 1,749,045 | Contig length | 1,784,933 |
|  | Total length of all alignments | 797,592 | Total length of all alignments | 775,958 |
|  | Average identity | 94.61 | Average identity | 91.86 |
|  | **Identical bases** | **754,602** | **Identical bases** | **712,795** |
| 2.1  2,105,923 | tig6^tct^ | | tig11867^tct^ | |
|  | Contig length | 2,096,600 | Contig length | 2,104,178 |
|  | Total length of all alignments | 2,010,934 | Total length of all alignments | 1,980,847 |
|  | Average identity | 94.64 | Average identity | 92.13 |
|  | **Identical bases** | **1,903,148** | **Identical bases** | **1,824,954** |
| 3.1^1^  1,700,800 | tig10^tct^ | | tig11878^tct^  MTLa | |
|  | Contig length | 1,950,477 | Contig length | 1,289,153 |
|  | Total length of all alignments | 1,631,051 | Total length of all alignments | 729,610 |
|  | Average identity | 94.1 | Average identity | 91.70 |
|  | **Identical bases** | **1,534,819** | **Identical bases** | **669,052** |
|  |  |  | tig00011870 |  |
|  |  |  | Contig length | 954,831 |
|  |  |  | Total length of all alignments | 859,421 |
|  |  |  | Average identity | 93.94 |
|  |  |  | **Identical bases** | **807,340** |
| 4.1  1,367,707 | tig11876^tct^ | | tig39^tct^ | |
|  | Contig length | 1,338,872 | Contig length | 1,386,788 |
|  | Total length of all alignments | 1,287,149 | Total length of all alignments | 1,267,915 |
|  | Average identity | 94.59 | Average identity | 92.07 |
|  | **Identical bases** | **1,217,514** | **Identical bases** | **1,167,369** |
| 5.1^1^  1,080,224 | tig11881^tct^  MTLalpha  rDNA | | tig11874^t^ | |
|  | Contig length | 1,095,871 | Contig length | 635,722 |
|  | Total length of all alignments | 1,013,327 | Total length of all alignments | 497,399 |
|  | Average identity | 95.41 | Average identity | 92.30 |
|  | **Identical bases** | **966,815** | **Identical bases** | **459,099** |
|  |  |  | tig00011878^tct^  MTLa | |
|  |  |  | Contig length | 1,289,153 |
|  |  |  | Total length of all alignments | 498,349 |
|  |  |  | Average identity | 91.60 |
|  |  |  | **Identical bases** | **456,488** |
| 6.1^1^  903,567 | tig29^tct^ | | tig16^tct^ | |
|  | Contig length | 1,749,045 | Contig length | 1,784,933 |
|  | Total length of all alignments | 634,361 | Total length of all alignments | 634,288 |
|  | Average identity | 94.7 | Average identity | 91.71 |
|  | **Identical bases** | **600,740** | **Identical bases** | **581,706** |
|  | tig3^ct^ | | tig1^tct^ | |
|  | Contig length | 2,603,961 | Contig length | 3,149,771 |
|  | Total length of all alignments | 216,182 | Total length of all alignments | 213,770 |
|  | Average identity | 94.46 | Average identity | 92.45 |
|  | **Identical bases** | **204,206** | **Identical bases** | **197,630** |
| 7.1  704,064 | tig11880^tct^ | | tig11884^tct^  rDNA | |
|  | Contig length | 1,017,382 | Contig length | 904,741 |
|  | Total length of all alignments | 655,192 | Total length of all alignments | 653,023 |
|  | Average identity | 93.79 | Average identity | 92.93 |
|  | **Identical bases** | **614,505** | **Identical bases** | **606,854** |
| 8.1  677,266 | tig64^tct^ | | tig60^tct^ | |
|  | Contig length | 798,529 | Contig length | 802,845 |
|  | Total length of all alignments | 592,388 | Total length of all alignments | 564,780 |
|  | Average identity | 94.52 | Average identity | 91.63 |
|  | **Identical bases** | **559,925** | **Identical bases** | **517,508** |
| All contigs | **Total alignment length across all contigs** | 11,684,935 | **Total alignment length across all contigs** | 11,473,415 |
|  | **Identical bases across all contigs** | 11,059,795 | **Identical bases across all contigs** | 10,587,038 |
|  | **Average identity across all contigs** | **94.65%** | **Average identity across all contigs** | **92.27%** |

^t^ Contains a telomere at one or both ends.

^c^ Contains a centromere.

^1^Where contigs resulted from a translocation between parts of two different contigs in the ATCC 96143 assembly, the sequence of the relevant contig was aligned to both contigs in the ATCC 96143 assembly. The average nucleotide identity for each alignment is shown separately. The average identity across all contigs was calculated as the total number of identical bases in all alignments divided by the total length of all alignments across all contigs. There appears to be an assembly error at the tip of scaffold 6 in the assembly of *C. metapsilosis* ATCC 96143 [(Oh *et al.* 2019)](https://paperpile.com/c/FunnkE/JKJW) (see Figure S2).
